# Anticancer and Antimicrobial Activities of Some Antioxidant-Rich Cameroonian Medicinal Plants

**DOI:** 10.1371/journal.pone.0055880

**Published:** 2013-02-11

**Authors:** Jean de Dieu Tamokou, Jean Rodolphe Chouna, Eva Fischer-Fodor, Gabriela Chereches, Otilia Barbos, Grigore Damian, Daniela Benedec, Mihaela Duma, Alango Pépin Nkeng Efouet, Hippolyte Kamdem Wabo, Jules Roger Kuiate, Augustin Mot, Radu Silaghi-Dumitrescu

**Affiliations:** 1 Department of Chemistry and Chemical Engineering, Babes-Bolyai University, Cluj-Napoca, Romania; 2 Tumor Biology Laboratory, The I. Chiricuta Oncology Institute, Cluj Napoca, Romania; 3 Department of Physics, Babes-Bolyai University, Cluj-Napoca, Romania; 4 Faculty of Pharmacy, Iuliu Hatieganu University of Medicine and Pharmacy, Cluj-Napoca, Romania; 5 State Veterinary Laboratory for Animal Health and Safety, Cluj-Napoca, Romania; 6 Department of Biochemistry, University of Dschang, Dschang, Cameroon; 7 Department of Chemistry, University of Dschang, Dschang, Cameroon; The University of Kansas Medical Center, United States of America

## Abstract

Traditional remedies have a long-standing history in Cameroon and continue to provide useful and applicable tools for treating ailments. Here, the anticancer, antimicrobial and antioxidant activities of ten antioxidant-rich Cameroonian medicinal plants and of some of their isolated compounds are evaluated.The plant extracts were prepared by maceration in organic solvents. Fractionation of plant extract was performed by column chromatography and the structures of isolated compounds (emodin, 3-geranyloxyemodin, 2-geranylemodin) were confirmed spectroscopically. The antioxidant activity (AOA) was determined using the 1,1-diphenyl-2-picrylhydrazyl (DPPH) bleaching method, the trolox equivalent antioxidant capacity (TEAC), and the hemoglobin ascorbate peroxidase activity inhibition (HAPX) assays. The anticancer activity was evaluated against A431 squamous epidermal carcinoma, WM35 melanoma, A2780 ovary carcinoma and cisplatin-resistant A2780cis cells, using a direct colorimetric assay. The total phenolic content in the extracts was determined spectrophotometrically by the Folin–Ciocalteu method. *Rumex abyssinicus* showed the best AOA among the three assays employed. The AOA of emodin was significantly higher than that of 3-geranyloxyemodin and 2-geranylemodin for both TEAC and HAPX methods. The lowest IC_50_ values (i.e., highest cytotoxicity) were found for the extracts of *Vismia laurentii, Psorospermum febrifugum, Pentadesma butyracea* and *Ficus asperifolia.* The *Ficus asperifolia* and *Psorospermum febrifugum* extracts are selective against A2780cis ovary cells, a cell line which is resistant to the standard anticancer drug cisplatin. Emodin is more toxic compared to the whole extract, 3-geranyloxyemodin and 2-geranylemodin. Its selectivity against the platinum-resistant A2780cis cell line is highest. All of the extracts display antimicrobial activity, in some cases comparable to that of gentamycin.

## Introduction

Many rural people in Cameroon still rely on plant-derived preparations for primary health care needs. In that respect, it appears useful to obtain a scientific basis for the possible use of herbal drugs in the treatment of diseases such as cancer, infectious diseases and those associated with oxidative damage. Here, we report the results of studies designed to evaluate the anticancer, antibacterial and antioxidant activities of selected Cameroonian medicinal plants, with previously demonstrated pharmacological activities. These plants were selected on the basis of recorded ethnobotanical knowledge, evidence for their continued wide usage and local availability.

The *Hibiscus asper* plant extract is an effective neuroprotective, with antioxidant and antiapoptotic activities [1], [2]. The *Tectona grandis* leaf extract was proven to be bioactive [3], due to the presence of two quinones: naphthotectone and anthratectone; it has antibacterial activity [4] and good antiradical properties [5]. A new compound: 5-hydroxylapachol [6], was isolated from the *T. grandis* root, and reported to be biologically active.

Extracts of *Ficus asperifolia* were tested on animal models [7], as well as on human cell cultures. The leaves extracts exhibit a moderate cytotoxic effect against breast cancer and malignant melanoma cell lines [8], enhance the normal human fibroblast growth and protect the normal cells against oxidative damage [9], thus conferring a good potential for selectivity for this plant extract.

*Rumex bequaertii* has a good biologic potential, having been tested as possible antiviral agent [10], while the *Pentadesma butyracea* fruit extracts have cytotoxic and antimalarial activities [11] due especially to cratoxylone, α-mangostin, 1,3,5-trihydroxy-2-methoxyxanthone, garcinone E, epicathechin and lupeol [12].

In the fruits and seeds of *Vismia laurentii*, the xanthones [13], anthraquinones [14], [15] and naphtoquinones are thought to be responsible for the anticancer [16] potential, as well as for the antimicrobial [17] and anti-malarial [18] activities of this plant extract.

The *Paullinia pinnata* crude extract is rich in triterpenoids [19] and displays a stimulatory effect on normal cell (fibroblast) proliferation. It is also an effective reactive oxygen radical scavenger due to its flavonoid, polyphenol and proanthocyanidin contents. Another effect linked to its antioxidant capacity is the ability to induce arterial relaxation [20].

*Dichrostachys glomerata* showed a promising antibacterial activity [21] due to the alkaloids, phenols and tannins content in the plant extract.

From *Psorospermum febrifugum* extracts, a series of xanthones [22] were isolated, which are responsible for its antiviral and anticancer activities in leukemia and colon cancer. The active constituents identified in this plant have the capacity to cause DNA damage, and to interact with a cytochrome *c*-viral DNA crosslink. Other constituents from the extracts were proven to be effective as antimicrobial agents [23]. One of these molecules is emodin [24], which is an effective antitumoral agent, with high efficacy against hepatic [25] and colon carcinoma [26], ovary [27], prostate [28], lung cancer [29] and other localizations. The compound is able to trigger apoptosis, inhibit the invasion and metastasis of malignant cells; it interacts synergically with the standard anticancer treatment, and can in fact reverse the drug resistance [30] of cancer cells. The anticancer and antioxidant activities of emodin and its two derivatives isolated from *Psorospermum febrifugum* stem bark were also investigated and reported herein.

## Materials and Methods

### Plant Material

Fresh aerial parts of *Ficus asperifolia* (stem bark), *Pentadesma butyracea* (fruits), *Psorospermum febrifugum* (stem bark), *Rumex abyssinicus* (bulbs), *Rumex bequaertii* (bulbs), *Paullinia pinnata* (leaves), *Tectona grandis* (leaves), *Hibiscus asper* (aerial part) and *Dichrostachys glomerata* (seeds) were collected from the West Region of Cameroon, and *Vismia laurentii* (Stem bark) was collected from Mbalmayo (Centre Region of Cameroon) in January-June, 2010, based on the traditional uses (Table 1). These plants were identified by Mr. Tadjouteu Fulbet of the Cameroon National Herbarium, where the voucher specimens were kept under the reference numbers (Table 1).

**Table 1:** Botanical identification, parts used, extraction solvent/yield and traditional therapeutic indications of medicinal plants studied.

Scientific name (Family)	Voucher specimen	Part used; extraction solvent and yield	Traditional therapeutic indications
*Vismia laurentii*De Wild (Guttiferae)	1882/SRFCam	Stem bark; CH_2_Cl_2_/MeOH1/1; 4%	Tonic, febrifugal, dermatitis, leprosy, scabies, eczemas, wounds [31]–[33].
*Ficus asperifolia* Miq. (Moraceae )	17054/HNC	Stem bark;MeOH;4%	Washing sores, ulcers, circumcision wounds [34], scraping patches of ringworm before further treatment [35], sterility/infertility, anthelmintic and purgative.
*Pentadesma butyracea Sabine* (Clusiaceae )	6861/SFRCam	Fruits; MeOH; 6%	Fever, coughs, constipation, bronchitis, and venereal diseases [36], [37], diarrhoea and dysentery.
*Psorospermum febrifugum*(Guttiferae)	36617/HNC	Stem bark; MeOH; 10.40%	Malaria, epilepsy, diarrhea [38]. Febrifugal, antidote against poison and purgative, leprosy, skin diseases (such as dermatitis, scabies and eczemas) and subcutaneous wounds [34], [39], [40].
*Rumex abyssinicus* (Polygonaceae)	27239/SRFCam	Bulbs; MeOH; 17%	Antiinflammatory, analgesic and antihelminthic [41].
*Paullinia pinnata* L. (Sapindaceae)	10702/SRFCam	Leaves; Hexane;6.71%	Typhoid, syphilis, gonorrhea, stomach-ache, waist pain, diarrhea, wounds [42], blood pressure, aphrodisiac.
*Tectona grandis* Linn. (Lamiaceae)	61993 HNC	Leaves; MeOH;16%	Bronchitis, biliousness, bronchitis, hyperacidity, dysentery, diabetes, leprosy, piles, leucoderma, expectorant properties, astringent, anthelmintic, inflammatory swelling, bilious headache and swellings [43], [44].
*Rumex bequaertii* De Wild (Polygonaceae)	7665/SRFCam.	Bulbs; MeOH; 14%	Furuncle, kwashiorkor, worms [45], cough.
*Hibiscus asper Hook.f. (Malvaceae)*	6970/SRF Cam	Aerial parts;Hexane;21.16%	Potent sedative, tonic and restorative, anti-inflammatory and antidepressive drug [1], pneumonia, typhoid and skin diseases
*Dichrostachys glomerata* (Forssk.) Chiov. (Mimosaceae)	15220/SRFCam	Seeds;CH_2_Cl_2_/MeOH 1/1;16.52%	Cough in children, gynecological disorders [46], antiviral, hypotension, wounds [47].

### Ethics Statement

For the collection of plants, no specific permits were required for the described field studies. For any locations/activities, no specific permissions were required. All locations where the plants were collected were not privately-owned or protected in any way and the field studies did not involve endangered or protected species.

### Extraction, Fractionation and Isolation

The air-dried and powdered sample from each plant was macerated separately in an organic solvent (Table 1) for 48 h at room temperature with occasional shaking. After 48 h, the extract was filtered through a Whatman no. 1 filter paper. The filtrate was then evaporated to dryness at 40°C (for dichloromethane) or 50°C (for hexane and methanol) under reduced pressure using a rotary evaporator to give a residue which constituted the crude extract. The extraction yield was calculated (Table 1) and the crude extract was kept at +4°C until further use.

The methanol extract of stem bark of *Psorospermum febrifugum* (520 g) was partitioned with EtOAc to afford 120 g of ethyl acetate extract. A part of the ethyl acetate extract (85 g) was subjected to vacuum liquid chromatography on silica gel, using hexane/ethyl acetate (from 15 to 100) resulting in 55 fractions of 200 mL each. After thin-layer chromatography monitoring, the following subfractions were obtained: fraction A (F_1–25_, 37 g), fraction B (F_26–45_, 32 g) and fraction C (F_46–55_, 13 g). Several column chromatography steps on fraction A, using hexane/ethyl acetate (from 2 to 60), yielded: 3-geranyloxyemodin (27 mg), 2-geranylemodin (312 mg), emodin (56 mg), friedelin (67 mg) and friedelannone (34 mg). Fraction B after several column chromatography steps (hexane/ethyl acetate, from 15 to 100), yielded betulinic acid (110 mg) and catechin (78 mg).

### Identification of the Isolated Compounds

The structures of the isolated compounds were established using spectroscopic analysis and by direct comparison with published information [48]–[50]. Melting points (uncorr.) were determined on a Kofler apparatus. The proton nuclear magnetic resonance and carbon 13 nuclear magnetic resonance were recorded at 500 MHz and 125 MHz respectively, with trimethylsilyl as an internal standard. Optical spectra were recorded with a NICOLET 510 P FT-IR spectrometer, a UV-2101 PC spectrometer, and a Perkin- Elmer 241 polarimeter. Silica gel 230–400 were used for flash chromatography and 70–230 mesh (Merck) were used for column chromatography, while percolated aluminum backed silica gel 60 F_257_ sheets were used for thin-layer chromatography, with different mixtures of hexane/ethyl acetate as eluents. Spots were visualized under ultra-violet light (254 nm) and (365 nm) or using a solution of methanol: sulfuric acid (1∶1) reagent followed by heating at 100°C.

#### Determination of total phenolic content

The total phenolic content was determined spectrophotometrically in the extracts by using the Folin–Ciocalteu method as previously described [51]. The Folin–Ciocalteu reagent was prepared by mixing 5 g sodium tungstate, 1.25 g sodium molybdate, 2.50 mL of 85% phosphoric acid, 10 mL 20% hydrochloric acid, 7.50 g lithium sulfate, two drops of bromine and deionized water to a final volume of 50 mL. Further, stock solutions of 20% sodium carbonate and 400 mg/l gallic acid were prepared. For each sample, 20, 10 and 1 µL of 10 mg/mL ethanolic extract or 20 µL of 1 mg/mL ethanolic isolated compounds were added to 640 µL distilled water and 200 µL freshly prepared Folin–Ciocalteu reagent, followed by incubation in the dark for 5 min. Then, 150 µL of 20% sodium carbonate solution were added and samples were incubated in the dark for 30 min. The solution turned deep blue. The final concentrations of the tested samples in the assayed solution were 100 µg/mL and 10 µg/mL for the extract and isolated compounds respectively. At the same time, gallic acid standards of 6.25, 12.50, 25, 50 and 75 µg/mL final concentration solutions were reacted with the Folin–Ciocalteu reagent in the same way as the samples. The UV-vis spectra of all the samples were recorded against the reference solution (zero gallic acid) and the absorbance was monitored at 725 nm. The measurements were done in triplicate. For the gallic acid standards, a calibration curve (Pearson’s correlation coefficient: *R* = 0.996) was constructed and the total level of phenolics for each sample was determined in terms of gallic acid equivalents.

### Antioxidant Assay

#### DPPH bleaching assay

The free radical scavenging activity of the extract as well as some of its isolated compounds was evaluated according to described methods [52]. Briefly, 10 µL of ethanolic extract/pure compounds were added in 990 µL of 20 mg/l ethanolic solution of DPPH (2, 2-diphenyl-1-picrylhydrazyl) found in the quartz UV-vis cuvette. The final concentrations of samples in the cuvette were 100 µg/mL for the crude extract and 10 µg/mL for the isolated compounds. Vitamin C was used as positive control at concentration of 10 µg/mL. The process was monitored spectrophotometrically at 517 nm for 136 s after sample addition. The percentages of the DPPH consumed until this point time of reaction were calculated according to formula (A_0s_ – A_136s_)/A_136s, blank_ where A refers to the 517 nm absorbance at 136 s reaction time and ‘blank’ refers to the sample to which ethanol was added instead of sample. Also, the area under the kinetic curve of DPPH bleaching was determined. This is a more appropriate parameter since is based on the whole kinetic profile rather than a single time point value [53].

#### Trolox equivalent antioxidant capacity (TEAC) assay

The TEAC test was done as previously described [54] with slight modifications. In a quartz cuvette, to 950 µL acetate buffer (pH = 5.0, 100 mM), the following were added: 20 µL pure laccase (1 µM stock solution), 20 µL test sample and 10 µL ABTS (2,2'-azinobis(3-ethylbenzothiazoline-6-sulfonic acid)) (74 mM stock solution). The laccase were purified from *Sclerotinia sclerotiorum* according to the protocol described [55]. The sample concentrations in the assay mixture were 200 µg/mL for the crude extract and 10 µg/mL for the isolated compounds. The content of the generated ABTS**^•+^** radical was measured at 420 nm after 230 s reaction time and was converted to gallic acid equivalents by the use of a calibration curve (Pearson’s correlation coefficient: *R* = 0.998) constructed with 0, 4, 10, 14, 28, 56, 70 µM gallic acid standards rather than Trolox. Experiments were done in triplicates.

#### Hemoglobin ascorbate peroxidase activity inhibition (HAPX) assay

Bovine hemoglobin was purified following the general protocol of Antonini and Brunori [56]. Hemoglobin ascorbate peroxidase activity has previously been described in detail [57], [58]. Stock solutions of met-hemoglobin, hydrogen peroxide and ascorbate of 1.4 mM (in PBS, pH 7.4), 50 mM (in water) and 70 mM (in water), respectively, were used. From the stock solutions, 7 µL of ascorbic acid were added to 948 µL of sodium acetate buffer (pH 5, 50 mM) in a quartz cuvette followed by the addition of 15 µL of hydrogen peroxide. After 20 s, 10 µL of ethanolic sample was added to the reaction mixture. All these processes were monitored spectrophotometrically at 290 nm. The kinetic profile after the addition of the plant extract samples remained linear for about 40 s. At 12–15 s after the addition of the plant extracts, 10 µL of met-hemoglobin from the stock solution was added to the reaction mixture and the 290 nm absorbance was further monitored for the inhibition of the consumption of ascorbic acid. A measurable significant inhibition of the ascorbic acid consumption was observed. For each sample, three measurements were run. The slope of each sample was calculated at the tested concentration and also without the tried sample (blank). The inhibition of the ascorbic acid consumption was determined as followed: HAPX = 100−[(slope of the sample/slope of the blank)×100].

Statistical analysis was carried out using Statistical Package for Social Science (SPSS, version 12.0). The experimental results were expressed as the mean ± Standard Deviation (SD). Group comparisons were performed using One Way ANOVA followed by Waller-Duncan Post Hoc test. A p value of 0.05 was considered statistically significant.

### EPR Measurements

The plant extract was dissolved in absolute ethanol; to 1000 µL of this solution, 5 µl of 10 M sodium hydroxide were added. The yellowish extract suddenly turned to a different color depending on the sample (hues of red, brown and green). Using a syringe and a needle, 500 µL of this brown solution were quickly transferred to an EPR tube and frozen at liquid nitrogen temperature. Equimolar ethanolic solutions of rutin, luteolin, hydroquinone, caffeic acid, gallic acid were separately treated with sodium hydroxide following the same protocol. The EPR spectra were measured using an EMX Micro spectrometer (Bruker BioSpin GmbH, Rheinstetten, Germany). EPR instrument conditions were: microwave frequency 9.43 GHz, microwave power 15.89 mW, modulation frequency 100 kHz, modulation amplitude 3 G, sweep rate 10 G/s; time constant 10.24 ms, temperature 100 K.

### Anticancer Assay

#### Cell lines and treatment

Human tumoral cell lines were used as biological models. Two lines were derived from skin cancer: A431 is the squamous epidermal carcinoma cell line (European Cell Culture Collection ECACC, no. 85090402) while the WM35 cell line is derived from melanoma, and it was generously offered by Prof. Falus Andras from Semmelweiss Institute Budapest, Hungary; the original source is ATTC [59]. Two ovary carcinoma lines were used: A2780 (ECACC no 91072201) and its cisplatin-resistant corresponding population: A2780cis cell line (ECACC no 93112517). The two skin cell lines were cultivated in Eagle’s Minimum Essential Medium-based nutrient, supplemented with L-glutamine, Non-Essential Aminoacids solution (NEA), Penicillin-Streptomycin solution and 10% Fetal Bovine Serum (FCS). Ovary cell lines A2780 and A2780cis were grown in an RPMI-1640 medium, supplemented with FCS, L-glutamine and antibiotics, in 25 mL culture flasks. All the cell lines were surface-adherent types. Cell cultures were kept in sterile incubators under 5% CO_2_, at 37°C. Subcultures were obtained by splitting the cell population at 70–80% confluency on flask, using 0.25% Trypsine solution with EDTA. All media and supplements were acquired from Sigma Aldrich Company; plastic disposables were from Nunc.

To perform the cell growth inhibition tests, cells were removed from flasks at subconfluency, and they were seeded on 96-well flat bottom culture plates, in 190 µl media, at 10^4^ cells/well concentration. After 24 hours, cells were treated with 10 µl solution from diluted plant extracts.

Normal human peripheral mononuclear leukocytes were obtained from whole peripheral blood. The sample was obtained by venipuncture from a 43-year female healthy donor, after her written informed consent. Lymphocytes were isolated with the gradient density method [60], using the Hystopaque 1077 solution (from Sigma Aldrich).

### Evaluation of Antiproliferative Capacity

The chemical cytotoxicity assays were performed on plant extracts obtained from *Hibiscus asper*, *Tectona grandis*, *Ficus asperifolia*, *Rumex abyssinicus*, *Rumex bequaertii*, *Pentadesma butyracea*, *Vismia laurentii*, *Paullinia pinnata*, *Dichrostachys glomerata*, *Psorospermum febrifugum* and 3 emodines isolated from *Psorospermum febrifugum*: 2-geranylemodin, 3-geranyloxyemodin and emodin.

Dried plant extracts and the purified compounds were diluted in absolute ethanol. Stock solutions concentrations varied between 95 µg/mL and 250 µg/mL, depending on solubility. Dilutions were made for every substance in Phosphate Buffered Saline Solution (PBS-Sigma Aldrich) to obtain a series of 7 dilutions for every substance. Final concentrations were between 0.50 µg/mL and 100 µg/mL for every substance, and considering that the maximum amount of substance/well was 10 µl, the final ethanol concentration on cells was 1%. The effect of solvent alone, without plant extracts/pure compounds, was verified and found to be negligible on the four cell lines.

For cytotoxicity assessment, a direct colorimetric assay, adapted from the Mossman MTT method was employed [61], [62] for the tumor cell lines. This cell viability assay is based on living cells’ property to transform the MTT dye tetrazolium ring into a purple-colored formazan structure due to the action of mitochondrial and other dehydrogenases inside the cell. The color intensity yielded by the cell population is directly proportional to the number of viable cells, and one can quantify the absorbance measurements using mathematical parameters. For the normal human lymphocytes, which are in suspension, the cytotoxicity was evaluated using the water-soluble MTS [63] dye (provided by Promega). Colorimetric measurements were performed on a BioTek Synergy 2 multiplate reader.

The 96-well plates containing the cell cultures were treated with serial concentrations of solutions of plant extracts/pure compounds, and the microplates were incubated under sterile conditions. Absorbance measurements were made after 24 h of incubation. Three independent experiments were performed for every cell line.

The results were analyzed using the Graph Pad Prism 5 biostatistics package, to obtain IC_50_ values for each compound from a dose-response nonlinear curve; in order to correlate the results, the one-way ANOVA method and Bonferroni post-test were used.

Validation of the MTT results was done by performing the resazurin viability assay (Alamar Blue test by Life Technologies Invitrogen). The test is based on quantitative measurement of the capacity of healthy living cell to maintain the cytoplasmatic reducing capacity. Resazurin is transformed into resorufin only inside the healthy cells, and the fluorescence yielded by this phenomenon was quantified by measurements using the above-described multiplate reader, set to 540/25 and 620/40 filters. Tumor cells were seeded on 96-well culture plates, and they were treated with 3 different concentrations from each extract. As reference, untreated cells were used. Concentrations were calculated to be identical with the IC50 and IC90 value for each compound. Every test was performed in duplicate. Linear regression analyses were performed using the GraphPad prism program.

We evaluated the proapoptotic potential of the extracts by an assessment of the phosphatidyl serine (PS) molecule translocation on cell surface during the apoptotic process. Alexa Fluor® 488 annexin V/Dead Cell Apoptosis Kit (provided by Life Technologies Invitrogen) was used. The test is able to distinguish living cells from apoptotic or necrotic ones, based on the different colorations which appear following the cell conjugation with Alexa Fluor labeled AnnexinV, and propidium iodide, respectively. Cells were treated for 12 hours with the plant extracts; the IC50- equivalent concentration was used for every cell line. Treated cells were harvested, processed according to the manufacturers protocol, and the stained cell population was disposed onto microscope slides. A Nikon Eclipse E600 inverted phase fluorescence microscope in conjunction with the Lucia software were employed in order to identify and count the green-coloured apoptotic cells, the red necrotic cells and uncolored living intact cells.

### Antibacterial Activity

The extracts were tested for antimicrobial activity at concentrations of 10 mg/mL against the Gram-positive strains *Staphylococcus aureus* (ATCC 4944) and *Lysteria monocytogenes* (ATCC 29211), and against the Gram-negative strains *Escherichia coli* (ATCC 25922) and *Salmonella typhymurium* (ATCC 14028), using the cup-plate agar diffusion method [64]. Each strain was suspended in Mueller Hinton (MH) broth at ∼10^6^ colony forming units/mL. This was then ’flood-inoculated’ onto MH agar and MH Dextrose AGAR surfaces, which were then dried. Six-millimeter diameter wells were cut from the agar using as sterile cork-borer, and 10 µL of each extract were delivered into the newly-created wells. This gave a charge of 100 µg of plant extract per well. The plates were incubated at 37°C and the diameters of the growth inhibition zones (in mm) were measured after 24 hours. Gentamycin (10 µg/well) was employed as standard antiobiotic where 40% and 70% aqueous alcohol solutions were used as negative controls. Results were obtained in triplicate.

## Results and Discussion

### Phytochemical Analysis

**Figure 1:**
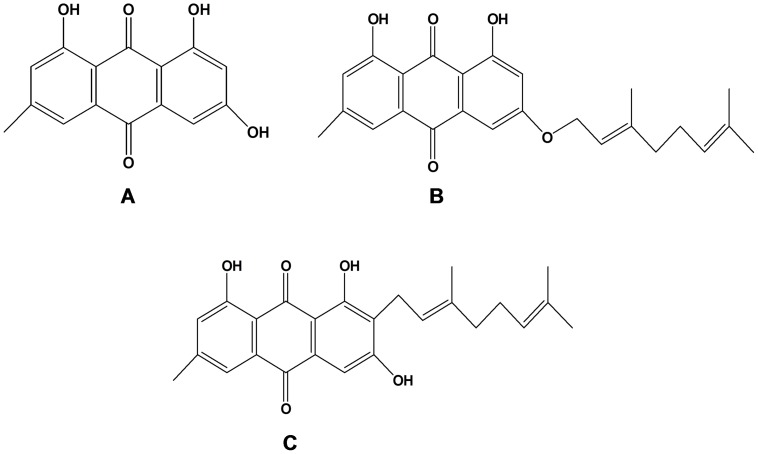
Chemical structures of the isolated compounds. The compounds isolated from *Psorospermum febrifugum* using column chromatography and identified using physicochemical and spectroscopic data were emodin (A), 3-geranyloxyemodin (B) and 2-geranylemodin (C).

Three known compounds: emodin (**1**), 3-geranyloxyemodin (**2**), 2-geranylemodin (**3**) were isolated from the CH_2_Cl_2_ : MeOH (1∶1) extract of *Psorospermum febrifugum* stem bark (Figure 1) and were tested for their antioxidant and anticancer activities.

#### Total phenol content

**Figure 2:**
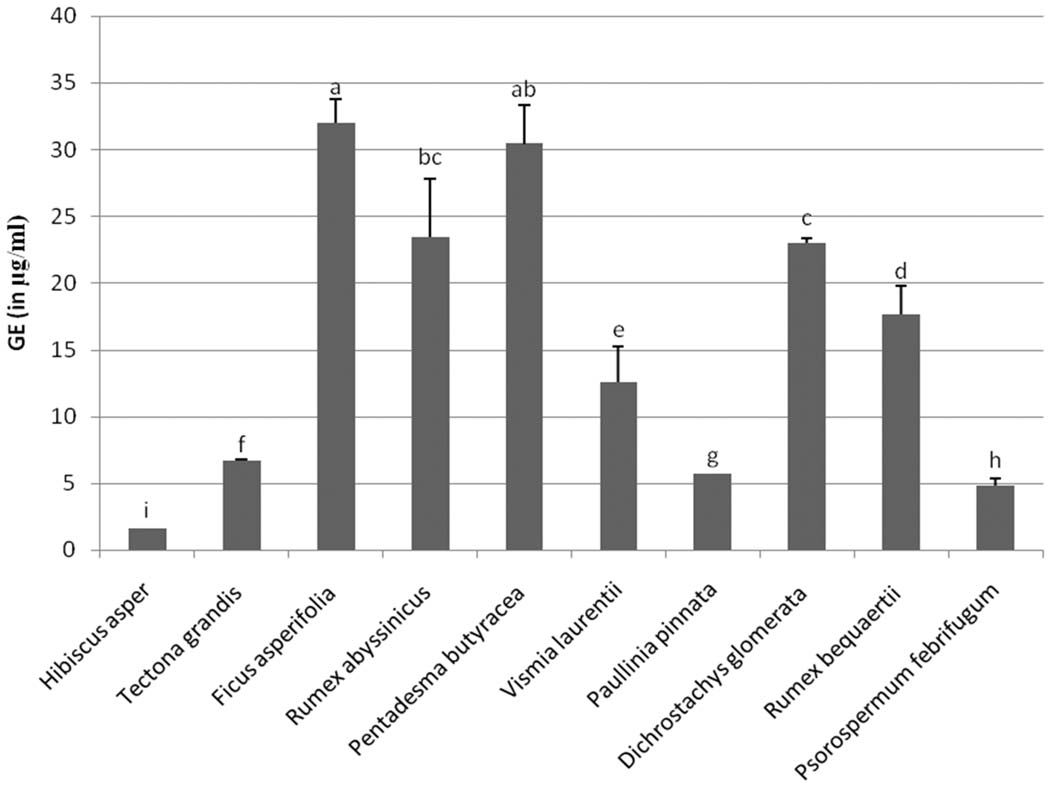
Total phenol content of the studied extracts. The total phenolic content is expressed as gallic acid equivalent (GE; µg/mL) in final plant extracts. Bars represent the mean ± SD of three independent experiments carried out in triplicate. Letters a–i indicate significant differences between samples according to one way ANOVA and Waller Duncan test; *p*<0.05.

The Folin-Ciocalteu assay is one of the oldest methods developed to determine the content of total phenols [65]. As shown in Figure 2, the total phenol content, expressed as gallic acid equivalents (GAE), was found to vary from 1.57 to 31.96 µg/mL in the extracts analyzed in the present work. The highest total phenol content was recorded with the MeOH extracts of *Ficus asperifolia* (GAE: 31.96 µg/mL) and *Pentadesma butyracea* (GAE: 30.43 µg/mL) while the hexane extract of *Hibiscus asper* (GAE: 1.57 µg/mL) contained the lowest amount of total phenols.

#### Antioxidant activity (AOA)

**Figure 3:**
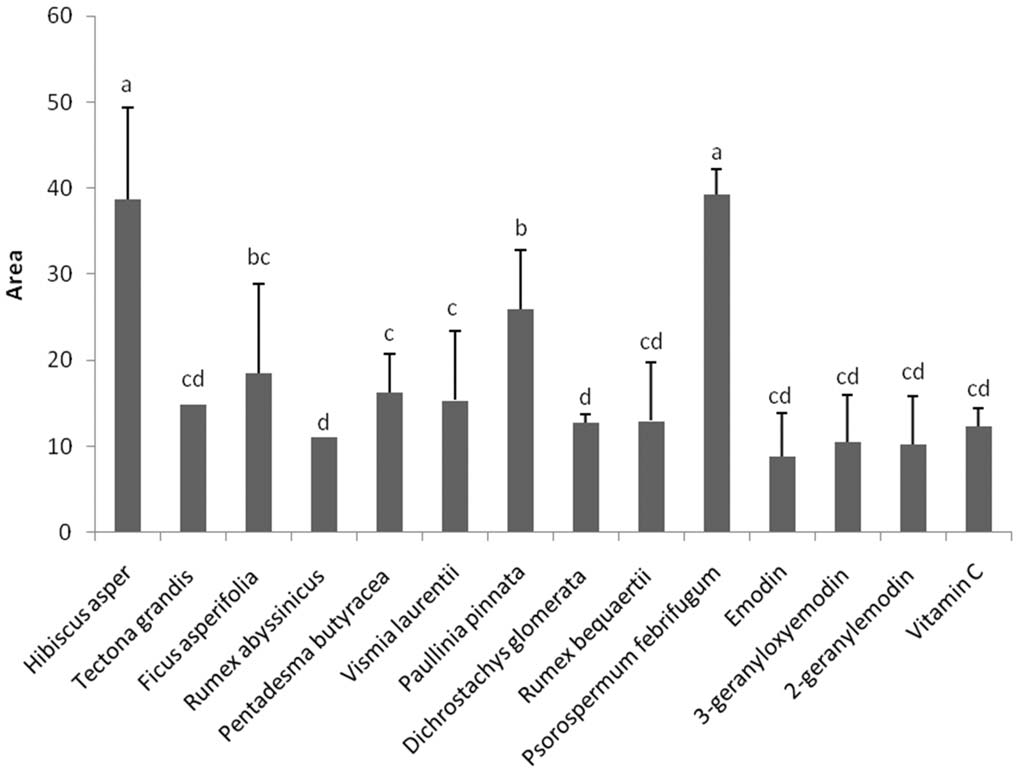
Antioxidant activity evaluated by DPPH method. Area under the kinetic curve of the DPPH bleaching by the test samples for the first 136 s. Bars represent the mean ± SD of three independent experiments carried out in triplicate. Letters a–d indicate significant differences between samples according to one way ANOVA and Waller Duncan test; *p*<0.05.

**Figure 4:**
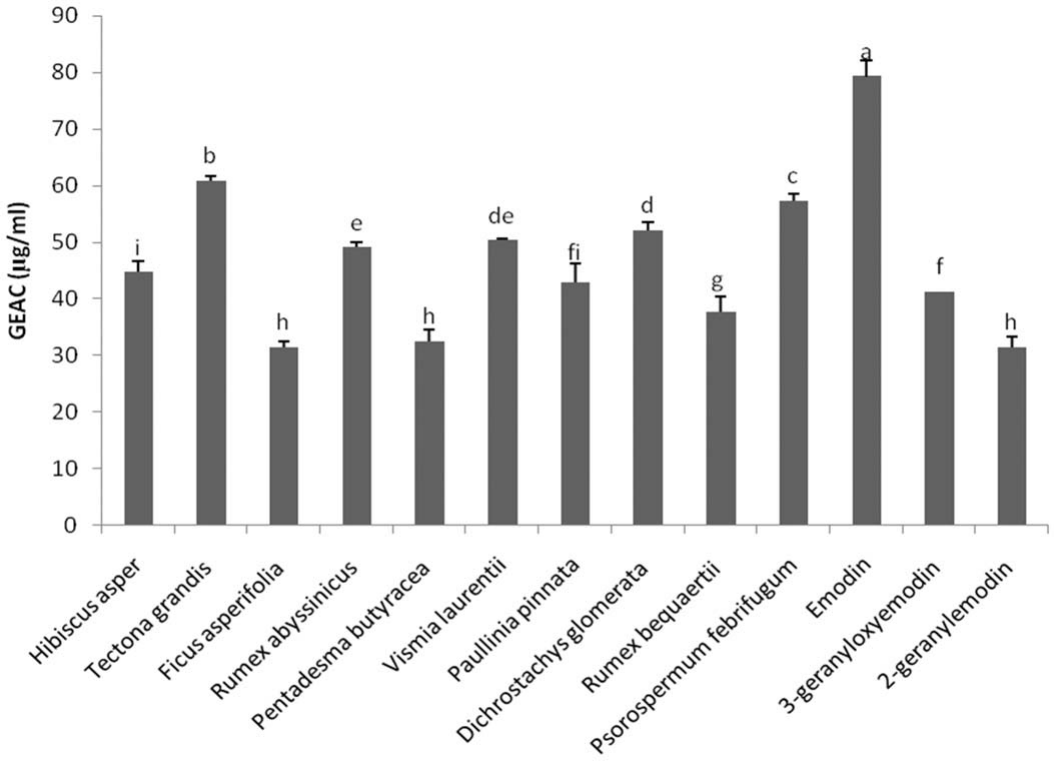
Antioxidant activity determined using TEAC method. Gallic acid was used as standard in place of trolox. Gallic acid equivalent antioxidant capacity (GEAC; µg/mL) of tested samples obtained by the TEAC procedure. Bars represent the mean ± SD of three independent experiments carried out in triplicate. Letters a-i indicate significant differences between samples according to one way ANOVA and Waller Duncan test; *p*<0.05.

**Figure 5:**
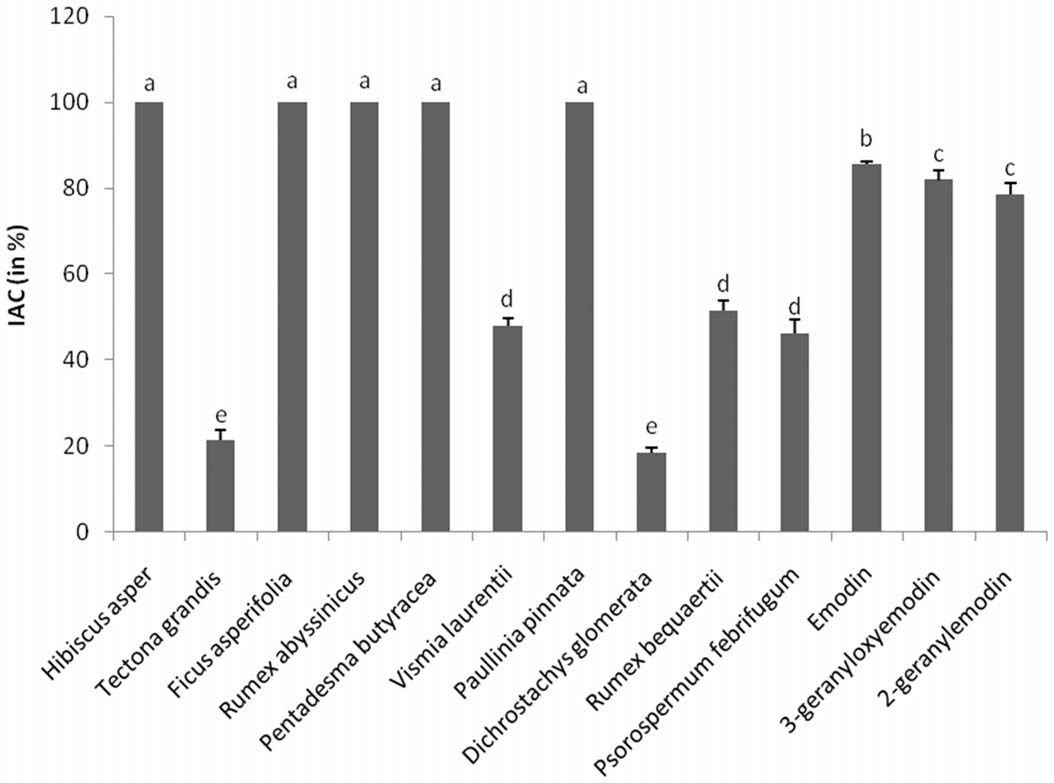
Antioxidant activity determined by the HAPX method. Inhibition of the ascorbic acid consumption (IAC in %) by the tested samples determined by the HAPX method. Bars represent the mean ± SD of three independent experiments carried out in triplicate. Letters a-e indicate significant differences between samples according to one way ANOVA and Waller Duncan test; *p*<0.05.

**Figure 6:**
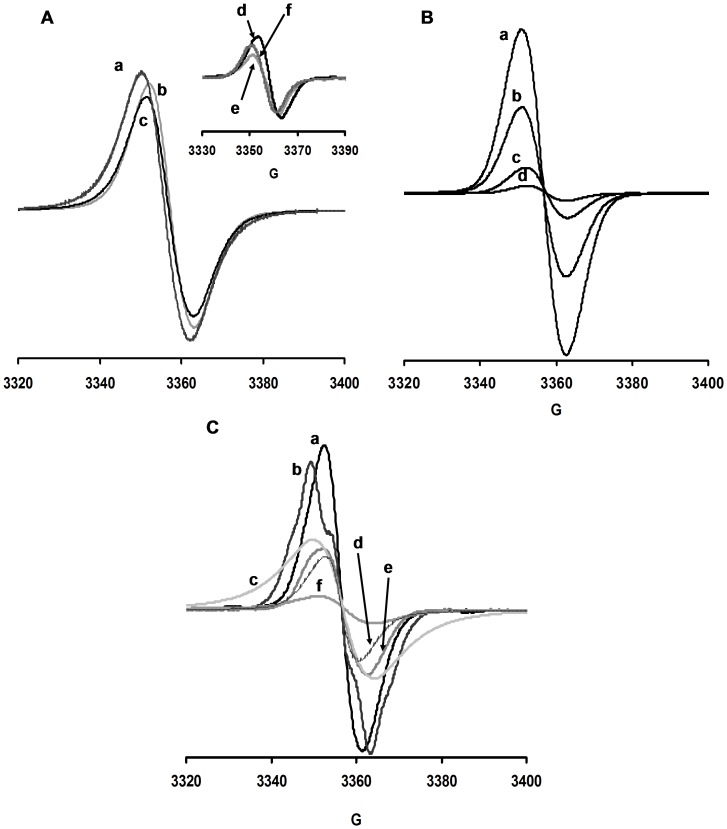
EPR spectra of the studied extracts. The EPR spectra of the ethanolic extracts of the studied plants were grouped according to shapes and/or intensity (panels A, B) and several phenolic references (C) treated with sodium hydroxide (final pH 12.7) for 15 s and measured at 100 K. **A.** a - *Rumex bequaertii*, b - *Rumex abyssinicus*, c - *Dichrostachys glomerata*, inset (y-axis scale is 100 times shorter): d - *Tectona grandis*, e - *Paullinia pinnata*, f - *Hibiscus asper*; **B.** a - *Pentadesma butyracea*, b - *Vismia laurentii*, c - *Ficus asperifolia*, d - *Psorospermum febrifugum*; **C.** a - luteolin, b - hydroquinone, c – caffeic acid, d - quercetin, e - rutin, f - gallic acid.

In this study, the AOA of the plant extracts and some of their isolated compounds were evaluated using three different methods - DPPH, TEAC and HAPX. The results obtained are summarized on Figures 3, 4, 5, 6. *Rumex abyssinicus* showed the best AOA among the three mechanisms used. For the DPPH method, the lowest area values corresponding to the most antioxidant substances were found for the extracts from *Tectona grandis, Rumex abyssinicus, Pentadesma butyracea, Vismia laurentii, Dichrostachys glomerata* and *Rumex bequaertii* while the highest area values (least activity) were those of *Hibiscus asper* and *Psorospermum febrifugum* (Figure 3). No significant differences were observed between the AOA of vitamin C (reference drug), emodin and its two derivatives (Figure 3).

For the TEAC method, the crude extracts of *Tectona grandis*, *Psorospermum febrifugum*, *Dichrostachys glomerata*, *Vismia laurentii* and *Rumex abyssinicus* showed the highest AOA (Figure 4). The least active extracts were those of *Ficus asperifolia* and *Pentadesma butyracea*. The AOA of emodin was significantly higher than those of 3-geranyloxyemodin and 2-geranylemodin for both TEAC and HAPX methods (Figures 4 and 5); their chemical structures (Figure 1) do not suggest significant differences in redox potentials (as also illustrated by values for a wide range of emodin derivatives, all of which are in the within −0.46 and −0.40 mV [66], hence suggesting that the hydrophobicity of the geranyl group does control the outcome of some antioxidant mechanisms (TEAC, HAPX, both accomplished in aqueous solution) - but not for the DPPH measurement (accomplished in ethanolic solution, where hydrophobicity would be less of a limitation than in water).

In addition to compounds **1**, **2** and **3,** phenolic derivatives were previously isolated from *Psorospermum febrifugum* [22], [24]. Phenolics are known to scavenge free radicals and active oxygen species such as singlet oxygen, superoxide anion radical and hydroxyl radicals [67], [68]. Therefore, the presence of such compounds could be partially responsible for the AOA found in this plant extract; the AOA depends the method used, reinforcing the concept that these extracts contain several antioxidant compounds that act in different manners.

*Hibiscus asper*, *Ficus asperifolia*, *Rumex abyssinicus*, *Pentadesma butyracea* and *Paullinia pinnata* were the most active plants for the HAPX method, while the least active were *Tectona grandis* and *Dichrostachys glomerata* (Figure 5). The AOAs of crude extracts from *Hibiscus asper*, *Tectona grandis*, *Ficus asperifolia* and *Paullinia pinnata* corroborate those of the literature [2], [3], [9], [69], [70]. To the best of our knowledge, this is the first systematic screening for the quantification of total phenolics and AOA of the crude extracts/compounds from *Dichrostachys glomerata*, *Psorospermum febrifugum*, *Pentadesma butyracea*, *Rumex abyssinicus*, *Rumex bequaertii* and *Vismia laurentii*.

#### Correlation between the antioxidant capacity and the total phenol content

The AOAs were weakly correlated with the total phenol content: *R* = –0.648 for the DPPH free-radical scavenging assay, indicating that phenolics partially participate in the DPPH scavenging, *R* = −0.600 for the TEAC method, showing that in samples with low phenolic content there are other extracted compounds responsible for ABTS^•+^ scavenging and in samples with high phenolic content there is little of this alternative type of compounds, and *R* = 0.224 for the HAPX assay showing that, at least for these samples, there are other types of compounds involved in this mechanism which are far more important than the phenolic compounds. These findings, and in particular the DPPH results (ethanolic solution protocol), are in line with our previous findings [71] but totally different than the HAPX and TEAC results (aqueous solution protocol) indicating once more that besides phenolics, in these extracts there are other types of compounds very important for antioxidant activity.

### EPR Results

**Figure 7:**
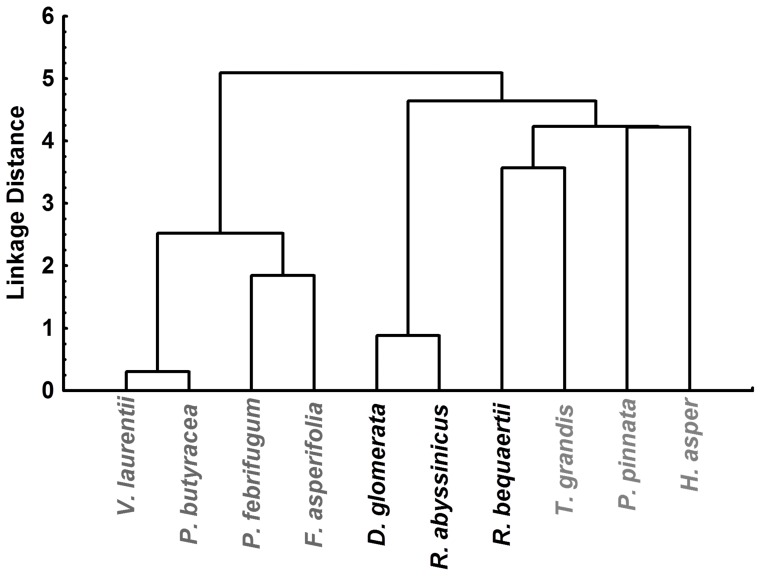
Cluster tree of the EPR spectra profiles. Dendogram is obtained using Ward’s linkage showing grouping of the plant extracts according to their EPR spectrum profiles. Group A: subgroup 1 *Rumex bequaertii*, *Rumex abyssinicus*, *Dichrostachys glomerata* (labelled in black), subgroup 2: *Tectona grandis*, *Paullinia pinnata*, *Hibiscus asper* (labelled in grey, on the right); Group B: *Pentadesma butyracea*, *Vismia laurentii*, *Ficus asperifolia*, *Psorospermum febrifugum* (labelled in grey, on the left) (details on EPR spectra profile in Figure 6).

It is known that treatment of phenolic compounds with alkali in aerobic conditions leads to stable semiquinone anionic radicals which can be detected using EPR spectroscopy. We have previously shown that treatment of natural extracts with a strong hydroxide solution leads to informative EPR signals which may vary in intensity and shape according to the chemical composition [71]. EPR spectra obtained in this manner for the studied extracts are plotted in Figure 6. Using cluster analysis (Ward’s method, using Statistica 8 software) applied on the first five factor scores obtained by applying PCA on the digitized EPR spectra which were previously normalized, according to the steps similarly described in [53], the spectra constitute two large groups: A (wide signals with various g values) and B (sharper signals with close g values but different intensities) (Figure 7). Group A can be further divided into two subgroups, based on their intensities (very high intensity and low intensity (inset in Figure 6)). Most probably, group B does not involve phenolic acids, p-quinoid structures similar to hydroquinone or glycosides of various phenolics since they have wider spectra (Figure 6C), but rather flavonoids such as luteolin and quercetin or other unidentified species. The difference in intensities may come both from phenolic content (content of radical generated) and chemical species (due to radical stability). Interestingly, the three isolated compounds yield very weak signals in EPR spectra, presumably due to a high instability and/or to a slow generation.

### Anticancer Activity

We evaluated the studied plant extracts activity against four malignant cell lines: A2780 and A2780cis ovary cell cultures, A421 epidermal carcinoma and MW35 melanoma cell cultures. Microscopic evaluation and colorimetric measurements were made after 24 hours of treatment with the tested substances.

**Figure 8:**
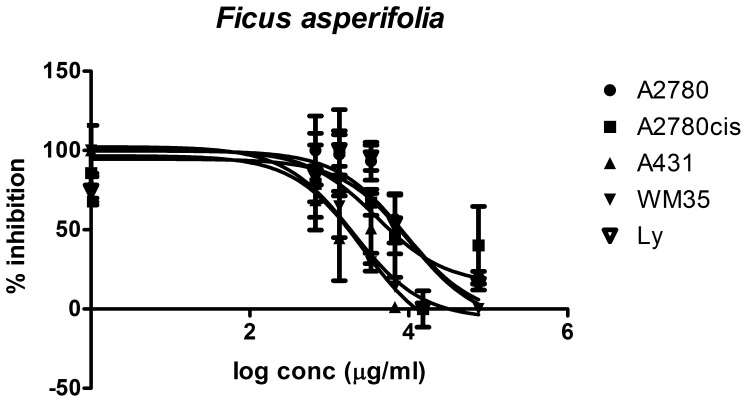
Cytotoxic effect of *Ficus asperifolia.* The cytotoxic effect was evaluated in four tumoral cells and is compared with their effect on normal human lymphocytes. Cells were treated in triplicate with eight different concentrations from the extract diluted in cell-friendly, non-toxic buffer, and the inhibitory effect against the treated cells proliferation was appraised after 24 hours.

**Figure 9:**
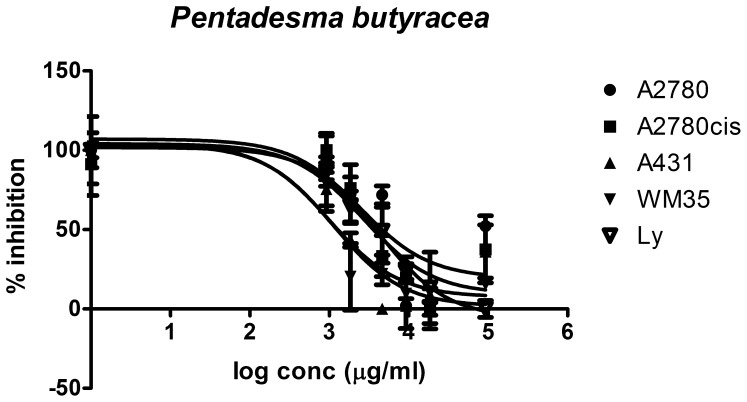
Dose response curve for *Pentadesma butyracea* **extract.** Sigmoidal dose-response curve corresponding to *Pentadesma butyracea* extract activity against four different cancer cell lines versus normal human lymphocytes.

**Figure 10:**
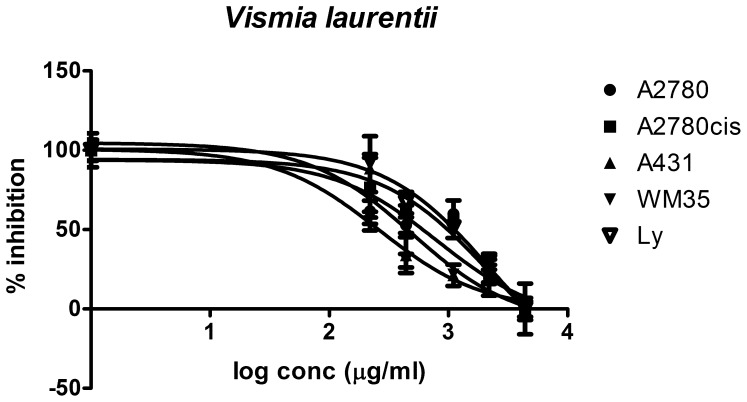
Cancer cell growth inhibitory activity of *Vismia laurentii* extract. Cancer cell growth inhibitory activity of *Vismia laurentii* extract against four different cancer cell lines.

**Figure 11:**
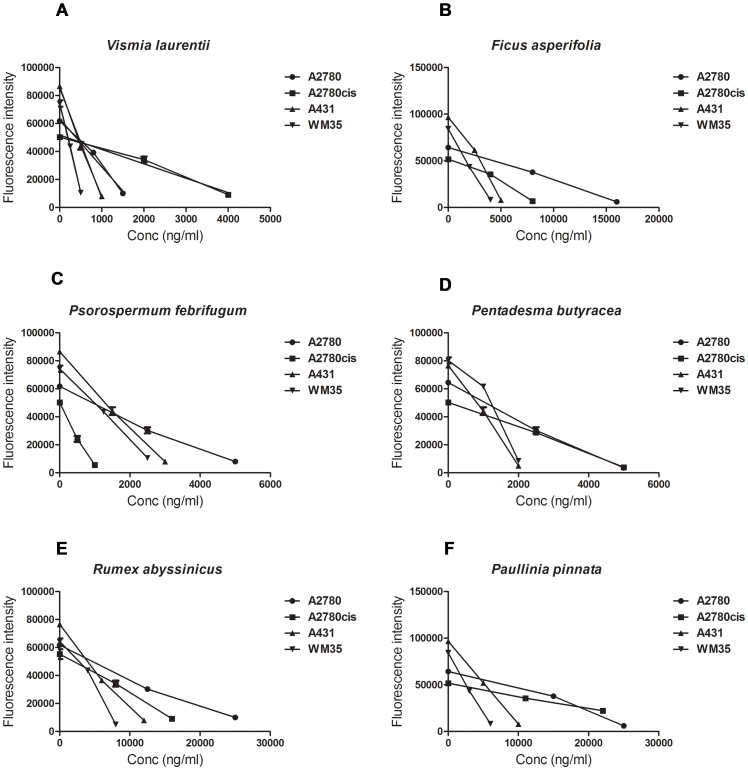
Cytotoxic activity for the tested extracts. The mitochondrial enzyme-based cytotoxic activity was validated with an independent cell viability test ruled on the reductive capacity of intact healthy living cells. Fluorescence measurements after a 24-hour exposure of the cells indicate a good correlation between the IC50 concentrations obtained by absorbance (colorimetric evaluation).

The ten plant extracts exhibit an inhibitory effect against tumor cell growth, with varying efficiencies and selectivities. The lowest IC_50_ values (corresponding to the most cytotoxic substances) were found for the extracts from *Vismia laurentii, Psorospermum febrifugum, Pentadesma butyracea* and *Ficus asperifolia* (Figures 8, 9, 10, 11). Some antiproliferative activity is also observed in *Rumex abyssinicus*, *Paulinnia pinnata*, *Tectona grandis*, *Hibiscus asper* and *Rumex bequaertii* extracts, but their IC_50_ values differ by an order of magnitude from the first group. The *Dichrostachys glomerulata* extract shows a much reduced activity, reflected by large IC_50_ values, and we can consider that this extract has no antiproliferative effect against cancer cells (Table 2).

**Table 2:** IC_50_ values (in ng/mL) of studied plant extracts against cancer cell lines and primary lymphocyte culture.

Plant extracts	A2780	A2780cis	A431	WM35	Ly
*Vismia laurentii*	811	2056	495	261	2139
*Ficus asperifolia*	8956	3968	2642	1991	10259
*Pentadesma butyracea*	2498	2704	1004	1128	4154
*Psorospermum febrifugum*	2633	359	1612	1280	2954
*Rumex abyssinicus*	12550	8014	6715	4612	9546
*Paullinia pinnata*	15220	22640	5311	3458	17190
*Tectona grandis*	20070	10075	2893	12680	15673
*Rumex bequaertii*	14440	29310	3615	22290	>50000
*Hibiscus asper*	29320	14650	35570	21550	>50000
*Dichrostachys glomerata*	243000	248500	>250000	>250000	>250000

**Figure 12:**
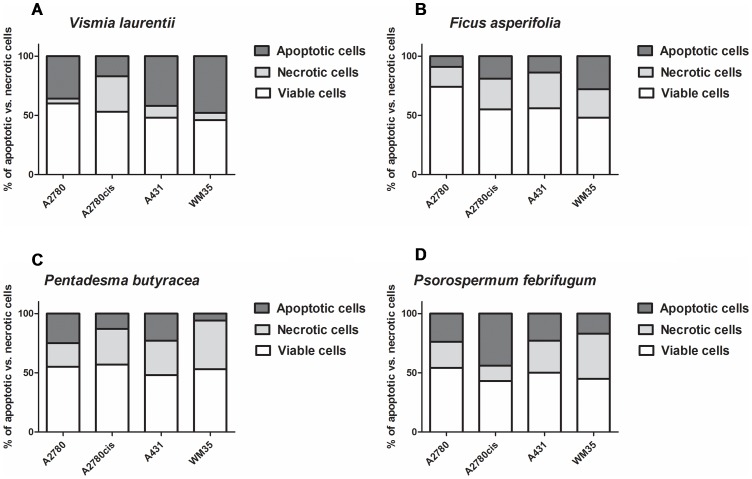
Apoptosis induction capacity of the natural extracts in treated cancer cell lines. For every cell line treated with the four active extracts, a number of 3×100 cells were evaluated microscopically. The distribution of apoptotic (colored in green due to the conjugation of PS with Annexin V-Alexa Fluor), necrotic (appears in red as a result of the intracellular PI coloration) and colorless intact viable cell percent is different for each compound. For every substance they are statistically significant differences between the activity against ovarian vs. epidermal cells (Two-way Anova and Bonferroni post-test in 95% confidence interval).

The *Ficus asperifolia* and *Psorospermum febrifugum* extracts are selective against A2780cis ovary cells, a cell line which is resistant to standard anticancer drug cisplatin. This tendency is observed also in *Rumex abyssinicus*, *Tectona grandis* and *Hibiscus asper* extracts, even if they are less effective against cell proliferation (Figures 12 A and B).

The resistant ovary cells display specific mutations of the p53 gene which alters its implication in the apoptotic pathway [72] and mitochondria targeting [73]. These resistant cells have a shorter doubling time compared to their sensitive counterparts [74]; their glutathione content is bigger, which favors resistance to platinum-based drugs but still leaves room for the action of natural compounds which may act on pathways different from those of the metal-based drugs. *Ficus asperifolia* extracts display an estrogenic effect [7]; *Psorospermum febrifugum* affect DNA strand breaks [22] which can constitute the basis of their excellent activity against the aggressive cisplatin-resistant cells. They can additionally act on PTEN, a tumor suppressor gene product, also overexpresed in resistant ovarian tumor cells [75].

The cytotoxic effect of *Ficus asperifolia* (Figure 8) as evaluated in four tumoral cells can be compared with the effect on normal human lymphocytes. Cells were treated in triplicate with eight different concentrations from the extract diluted in cell-friendly, non-toxic buffer, and the inhibitory effect against the treated cells proliferation was appraised after 24 hours (see also Table 2). *Ficus asperifolia* action is dose-dependent in all cell lines, the extract activity is pronounced against the epidermal cell lines, and IC50 values is significantly lower in A2780cis, A431 and WM35 cell lines, denoting an increased toxicity compared to normal lymphocytes. The extract displays a notable ability to distinguish between chemoresistant and chemosensitive ovary cells, the antiproliferative activity being much higher in the A2780cis cells. There are no disparities between the extract’s action against A2780 cell line and lymphocytes.

Most of the examined extracts inhibit the growth of A431 epidermal carcinoma cells, except *Hibiscus asper* and *Dichrostachys glomerulata*. The aggressive malignant melanoma cell WM35 growth is inhibited successfully by *Vismia laurentii*, *Ficus asperifolia*, *Pentadesma butyracea*, *Psorospermum febrifugum* and *Paulinnia pinnata* extracts (Figures 12 C and D).

Differences between extract activity against A2780 cells are very significant (one-way ANOVA test, Bonferroni post-test, p<0.05), the only exception being the fact that there are no significant differences between *Pentadesma butyracea* and *Psorospermum febrifugum*, and between *Paullinia pinnata* and *Rumex bequaertii* (Figures 12 A-D). In the 95% confidence interval, there are significant differences between all the extracts’ IC_50_ values in the biological evaluation on A2780*cis* cells. The same tendency is observed in the A431 and WM35 malignant skin cells (Figures 12 C and D).

The A431 carcinoma cell line also displays p53 mutations and the mitochondrial membrane potential down-regulation is possible by the active compounds of the plant extracts. The WM35 cell line is p53 wild-type, it expresses functional PTEN [76] and its growth inhibition is inhibited by other extracts, while the triggering of apoptotic pathway is different from the other three cell lines.

**Figure 13:**
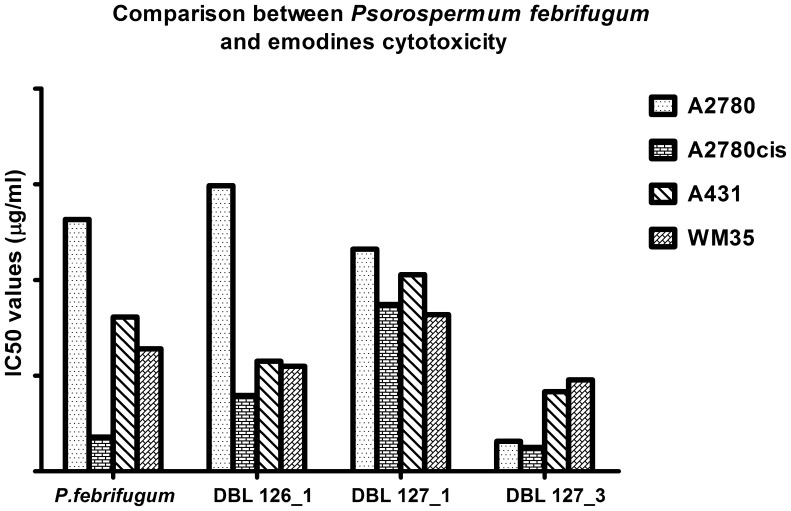
Comparison between *Psorospermum febrifugum* extract and emodines cytotoxicity. Differences between anticancer activity of *Psorospermum febrifugum* extract and its isolated compounds against four different cancer cell lines.

We also analyzed three pure compounds derived from *Psorospermum febrifugum* crude extract: emodin (**1**), 3-geranyloxyemodin (**2**) and 2-geranylemodin (**3**) (Table 3 and Figure 13). The three compounds display toxicity against cancer cells; the pattern of IC_50_ values distribution is different from the plant extract: emodin is more toxic compared to the whole extract, but less selective. 3-geranyloxyemodin is less toxic against the platinum-resistant A2780cis cell line and against dermal cell lines, and to some extent more toxic against A2780 cells (differences are statistically not significant). The activity of 2-geranylemodin is superior to that of the *P. febrifugum* extract in epidermal cell lines, and its antiproliferative capacity is slightly decreased in ovary cell lines, but the selectivity against the cisplatin-resistant A2780cis cells is maintained. These results illustrate similarities with the literature data as regards anticancer activity of emodines isolated from other plants [25]–[30]. Emodines separated from plant extract are responsible for the cytotoxicity of *P. febrifugum*, but probably they are not the only active constituents of this plant extract.

**Table 3:** IC_50_ values (in µg/mL) of *Psorospermum febrifugum* extract and its isolated compounds against four different cancer cell lines.

Extract/compounds	A2780	A2780cis	A431	WM35
*Psorospermum febrifugum*	2633	359	1612	1280
2-geranylemodin	2984	792	1150	1098
3-geranyloxyemodin	2321	1742	2056	1637
Emodin	314	250	832	957

The same pattern of antiproliferative potential of the six most active extracts: *V. laurentii, P. febrifugum, P. butyracea, F. asperifolia, R. abyssinicus* and *P. pinnata* wa*s* observed by analyzing the treated cells reduction potential (Figure 11). The liniar regression indicates r^2^ values between 0,95 and 0,99 as goodness of fit in 95% confidence interval, and in every case the slope was significant different from zero, with p<0,0005 for all cell lines.

The extracts of *V. laurentii, F. asperifolia*, *P. febrifugum, P. butyracea,* which display the highest selectivity between the action against tumoral cells and normal lymphocytes, are able to induce early apoptotic processes in treated cells (Figure 12), and this mechanism will conduct later to a massive cell loss. In two cases the action against cisplatin-resistant ovary cells was even more aggressive as for the sensitive ovary carcinoma cells, due to the faster translocation of the PS on the cell membrane, which activates the signaling pathway for cell recognition and immune reaction of macrophages in the human body.

Interestingly, the classification of the samples based on the EPR profiles correlates well with the cytotoxic results. Samples in group A have high IC_50_ values, while group B chas low IC_50_ (Table 2 and Figure 7). This indicates that the compounds responsible for this activity are phenolics and that they can generate stable semiquinone anionic radicals similar in structure and kinetics.

Our results agree with the literature data regarding *Vismia laurentii* [16], *Psorospermum febrifugum* [25]–[27] and *Ficus asperifolia* [8] anticancer potential, and we additionally identify the *Pentadesma butyracea* extract as a good antiproliferative agent, after an earlier report on its antimalarial activity [11]. We identify here two new targets: ovary cancer and skin malignancies, in which the prodrug potential of the natural substances can be used as a new therapeutic approach.

### Antimicrobial Activity

The plant extracts examined in the present study all display antimicrobial activity levels promising useful applications against representative strains (Table 4). Particularly, the *R. abyssinicus* extract displays an activity comparable to that of gentamycin, and *P. butyracea* extract follows closely. The antibacterial activities of crude extracts from *P. febrifugum, D. glomerata*, *V. laurentii, R. abyssinicus, F. asperifolia* and *T. grandis* corroborate those of the literature [3], [9], [16], [20], [22]. However, the antibacterial activities of the crude extracts from *H. asper* and *P. Butyracea* are reported here for the first time.

**Table 4:** Antimicrobial activities (diameters of growth inhibition zones in mm) of plant extracts examined in the present study.

Extract	*Salmonella typhymurium*	*Listeria monocytogens*	*Escherichia coli*	*Staphylococcus aureus*
Control: 70% alcohol	0	0	0	0
Control: 40% alcohol	0	0	0	0
Reference: gentamycin	18	18	22	19
*H. asper*	11	11	11	16
*T. grandis*	12	14	15	12
*F. asperifolia*	8	12	12	12
*R. abyssinicus*	15	25	21	18
*P. butyracea*	16	16	15	18
*V. laurentii*	10	12	8	10
*D. glomerata*	13	16	14	18
*P. febrifugum*	6	12	6	10

### Conclusion

The overall results of the present study provided evidence for the antioxidant, anticancer and antimicrobial activities of studied plant extracts/isolated compounds, and bring supportive data for future investigations that will lead to their use in cancer, oxidative stress and antimicrobial therapy.
